# Lipid-Coated Zinc Oxide Nanoparticles as Innovative ROS-Generators for Photodynamic Therapy in Cancer Cells

**DOI:** 10.3390/nano8030143

**Published:** 2018-03-02

**Authors:** Andrea Ancona, Bianca Dumontel, Nadia Garino, Benjamin Demarco, Dimitra Chatzitheodoridou, Walter Fazzini, Hanna Engelke, Valentina Cauda

**Affiliations:** 1 Department of Applied Science and Technology, Politecnico di Torino, Corso Duca degli Abruzzi 24, 10129 Turin, Italy; andrea.ancona@polito.it (A.A.); bianca.dumontel@polito.it (B.D.); nadia.garino@polito.it (N.G.); s223910@studenti.polito.it (W.F.); 2 Center for Sustainable Future Technologies—CSFT@POLITO, Istituto Italiano di Tecnologia, Corso Trento 21, 10129 Turin, Italy; 3 Department of Chemistry, Ludwig-Maximilians-University of Munich, Butenandtstrasse 11E, 81377 Munich, Germany; benjamindemarcov@gmail.com (B.D.); dimitra.ch93@googlemail.com (D.C.)

**Keywords:** zinc oxide nanoparticle, supported lipidic bilayer, reactive oxygen species, electron paramagnetic spectroscopy, photodynamic therapy, colloidal stability, 5,5-dimethyl-l-pyrroline-*N*-oxide (DMPO)

## Abstract

In the present paper, we use zinc oxide nanoparticles under the excitation of ultraviolet (UV) light for the generation of Reactive Oxygen Species (ROS), with the aim of further using these species for fighting cancer cells in vitro. Owing to the difficulties in obtaining highly dispersed nanoparticles (NPs) in biological media, we propose their coating with a double-lipidic layer and we evaluate their colloidal stability in comparison to the pristine zinc oxide NPs. Then, using Electron Paramagnetic Resonance (EPR) coupled with the spin-trapping technique, we demonstrate and characterize the ability of bare and lipid-coated ZnO NPs to generate ROS in water only when remotely actuated via UV light irradiation. Interestingly, our results reveal that the surface chemistry of the NPs greatly influences the type of photo-generated ROS. Finally, we show that lipid-coated ZnO NPs are effectively internalized inside human epithelial carcinoma cells (HeLa) via a lysosomal pathway and that they can generate ROS inside cancer cells, leading to enhanced cell death. The results are promising for the development of ZnO-based therapeutic systems.

## 1. Introduction

In recent years, major efforts have been devoted to the development of novel suitable nanotechnological platforms to improve medical diagnosis and therapies. Thanks to their higher area to volume ratio compared to larger particles, several nanomaterials have been exploited to deliver drugs specifically towards target regions or to elicit cytotoxic effects only when externally stimulated [1]. Among these, nanostructured zinc oxide (ZnO) has been widely studied, due to its interesting properties, such as piezoelectric and pyroelectric behaviors and biocompatible features, being classified as a “GRAS” (generally recognized as safe) substance by the Food and Drug Administration (FDA) [2,3]. As a semiconductor, it shows a band gap of 3.3 eV, thus it can adsorb ultraviolet (UV) light and emit it in the visible range, showing potential capabilities as light emitting diode (LED) or as a reporter in bio-imaging applications. The optical properties of ZnO can be efficiently exploited for the generation of Reactive Oxygen Species (ROS). Actually, in the presence of a radiation having a wavelength of less than 400 nm, the electrons (e^−^) are excited from the valence (VB) to conduction band (CB), generating positive holes (h^+^). In aqueous environment, the photo-generated e^−^ can reduce oxygen molecules, forming superoxide radical anion (O_2_^−^) [4,5], while the h^+^ can oxidize water molecules and hydroxide ions, generating hydroxyl radicals and hydrogen peroxide (H_2_O_2_) molecules [6]. Furthermore, the recombination of the electron–hole pair can generate emission of a photon (radiative recombination), which in turn can excite ground state oxygen generating singlet oxygen. All these ROS, when produced intracellularly, can exert highly cytotoxic effects [7] and one can take advantage of their generation for killing tumor cells. Actually, an overproduction of intracellular ROS produces oxidative stress [8], altering the cell cycle [9] and promoting cell death through apoptosis [5] or autophagy [10]. Moreover, ROS can induce lipid peroxidation, associated with impairment of cell membrane structure [11], protein denaturation and different types of DNA damage [12].

Therefore, several studies have proposed the photoexcitation of nanostructured ZnO to produce intracellular ROS as an effective therapeutic strategy, called Photodynamic Therapy (PDT), causing severe toxicity in different cancer cell lines [13,14]. This therapy promises better selectivity and fewer side-effects compared to most traditional chemo- and radio-therapies. ZnO nanoparticles (NPs) can indeed accumulate specifically within the tumor region thanks to the Enhanced Permeation and Retention effect [15]. In this way, when the light is directly focused in the region of interest, the therapeutic effect is highly localized. However, the PDT shows limited tissue penetration owing to the small penetration depth of UV light (less than 1 mm) used to excite the NPs. Therefore, PDT can be efficiently exploited for superficial tumors or with optical wave-guide irradiation in the case of deeper but accessible cancer tissues.

Using the Electron Paramagnetic Resonance (EPR) technique, the ROS species generated by ZnO NPs in aqueous solutions can be efficiently recognized [16]. It was reported that the ROS generated and thus the PDT effects are size dependent in the range from 20 to 100 nm [17], where smaller NPs present higher cytotoxicity. Further efforts are thus needed to unravel the effective production mechanism of ROS from nanostructured ZnO, in particular when the ZnO particle surface is modified by other molecules, thus functionalized.

According to the above considerations, the present paper reports on the preparation of ZnO NPs of about 20 nm in diameter and their surface functionalization to improve their biological stability and biocompatibility. The final aim is to study the effect of the surface molecular groups on the final ROS production and species. A self-assembly of phospholipids was achieved forming a supported lipid bilayer on the surface of the bare ZnO NPs. The results show that highly stabilized NPs without aggregation are obtained in Phosphate Buffered Saline (PBS) when the lipidic bilayer is formed. A deep investigation through the EPR technique is here proposed on the ROS produced under UV irradiation by bare and lipid-coated NPs in different aqueous, physiological and biological fluids. The results reveal that there is an important variability of the ROS production types depending on the NPs surface chemistry and dispersion media. The internalization into HeLa cancer cells and the intracellular ROS production are studied. Finally, the cytotoxic activity of both bare and lipid-coated NPs at different concentrations and under UV-stimulus activation is evaluated, leading to efficient cancer cell death. Therefore, the reported nanosystem, conjugating high stability in biological media and remote-activated cytotoxicity, can be a novel powerful tool to nanomedicine therapy against cancer.

## 2. Results and Discussion

### 2.1. ZnO Nanoparticles Synthesis and Characterization

**Figure 1:**
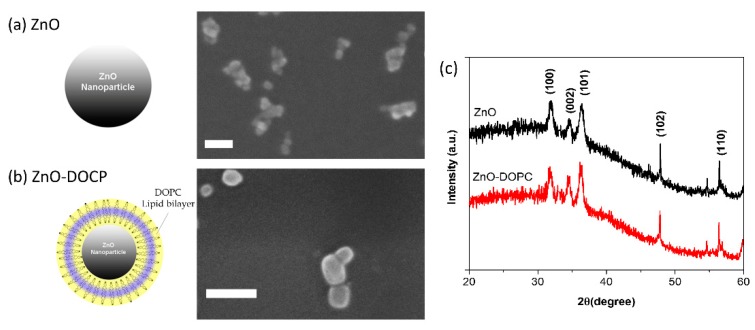
Morphology and crystalline structure of bare and lipid-coated zinc oxide NPs. Scheme and FESEM images of: bare ZnO NPs (**a**); and lipid-coated ZnO NPs (**b**). For FESEM images, all the NPs were coated by a thin layer of Pt. Scale bare is 30 nm in both images. (**c**) Representative X-ray diffractograms of the ZnO NPs: pristine (black curve) and lipid-coated (red curve) ones. Non-indexed peaks derive from the silicon wafer used as sample substrate.

ZnO nanoparticles were synthesized by a simple wet chemical method, as reported in detail in the Materials and Methods Section. The morphology and particle size of the as-prepared ZnO nanoparticles were characterized by Field Emission Scanning Electron Microscopy (FESEM), as shown in Figure 1a. The FESEM image shows that these nanoparticles have a spherical shape with an average diameter of 14 ± 2 nm (as measured from FESEM images using Fiji software, Open source, *n* = 50). Analysis of the X-ray diffraction pattern confirmed the crystalline structure of the ZnO NPs. The diffraction peaks matched well with the characteristic peaks of the single-phase wurtzite crystalline structure, as shown in Figure 1c (black curve). Applying the Debye–Scherrer equation to the broad diffraction peaks, an average size of 15 nm of the nanocrystallites was obtained, in fair agreement with the electron imaging results.

The lipid-coated nanoparticles were prepared by a solvent-exchange method using the commercial phospholipid DOPC (1,2-dioleoyl-sn-glycero-3-phosphocholine). As shown in Figure 1b,c, Field Emission Scanning Electron Microscope (FESEM) and X-ray Powder diffraction (XRD) analyses confirmed that the morphology and the crystalline structure of the NPs was not modified after the functionalization, except in the size of lipid-coated ZnO NPs (average diameter of 21 ± 5 nm, measured from FESEM images using Fijisoftware, *n* = 20).

**Figure 2:**
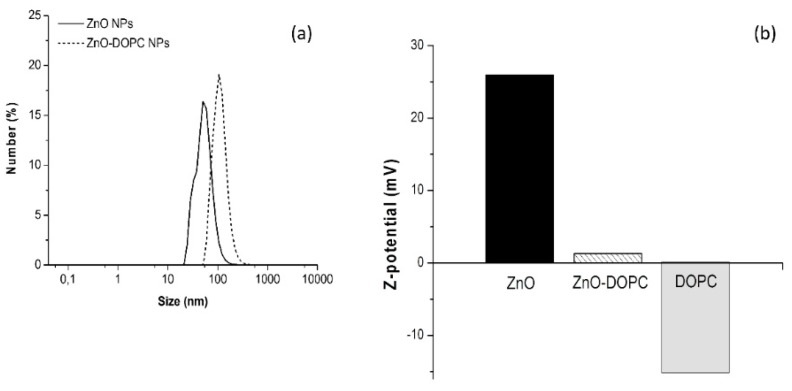
Hydrodynamic diameters and Z potentials of bare and lipid-coated ZnO NPs. Dynamic Light Scattering (**a**); and Z-potential (**b**) measurements of the two samples compared to DOPC lipids micelles.

Dynamic Light Scattering (DLS) experiments were performed for the two samples to evaluate their hydrodynamic diameters and stability in water (Figure 2a). The absence of micrometer-scale aggregates in all measurements suggests good dispersion and low aggregation behavior for both samples. Interestingly, the lipidic functionalization of the ZnO nanoparticle surface contributed to a larger mean hydrodynamic diameter (110 nm for ZnO-DOPC NPs) than the one obtained for the pristine ZnO NPs (55 nm).

Moreover, the Z-potential of lipids micelles and of bare and lipid-coated ZnO NPs was evaluated in water maintaining neutral pH by titration with NaOH and HCl 1 M. As shown in Figure 2b, the DOPC micelles present a negative Z potential, equal to −15 mV. The obtained value is in fair agreement with literature studies, that attribute it to a characteristic orientation of lipids polar head in solutions with low ionic strength [18].

Concerning the nanoparticles samples, different values were obtained depending on the surface properties of the ZnO NPs. For pristine ZnO NPs, the measured positive Z-potential (26 mV) is in good agreement with the literature values [19] and it is due to the protonation of the hydroxyl groups at the nanoparticle surface. A strong decrease of the Z-potential value was obtained for lipid-coated ZnO nanoparticles: the DOPC phospholipid shell neutralized the positive charges of the ZnO surface and lowered the Z-potential down to 1.3 mV. Showing different behaviors for pristine and functionalized ZnO nanoparticles, the DLS and Z-potential measurements together clearly suggest that the lipid functionalization worked successfully, almost completely shielding the ZnO NP in a protective lipid shell.

**Figure 3:**
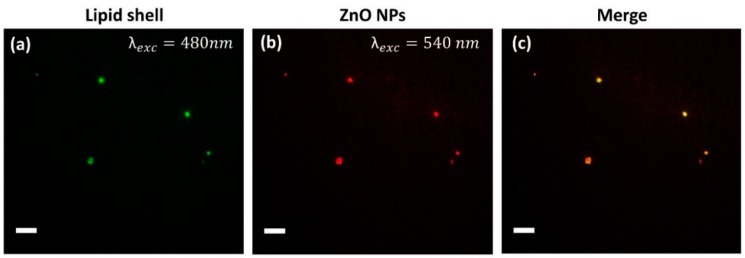
Co-localization of the lipid-shell with ZnO NPs. Wide-Field Fluorescence images of: (**a**) lipid-shells labeled by 1% Bodipy-DHPE; (**b**) amino-propyl functionalized (ZnO-NH_2_) nanoparticles marked with Atto550-NHS ester; and (**c**) the merged images showing the co-localization of the lipid-shell with the ZnO NPs. Scale bar: 10 µM.

To further confirm the formation of the lipid bilayer on the ZnO NPs surface, fluorescence microscopy co-localization experiments in wide-field configuration were performed (Figure 3). The DOPC shell was marked with 1% Bodipy FL DHPE lipid, characterized by a fluorescent excitation maximum at 488 nm, while ZnO NPs, functionalized with amino-propyl groups, were marked with Atto550-NHS ester dye, having a fluorescence excitation at 550 nm. Merging the two distinct images, green (lipid shell) and red (ZnO NPs) spots overlap forming a yellow spot corresponding to the co-localized lipid shell on the nanoparticle surface, thus further confirming the successful coverage by phospholipids of the ZnO nanoparticles. 

It is worth mentioning that the scale bar in the optical fluorescence images of Figure 3 is 10 µM. Thus, the resulting size of the nanoparticles can seem in disagreement with the sizes measured by DLS and FESEM. However, one should note that the magnification used for fluorescence imaging (in this case, 60× objective) is not enough to resolve sizes of 20 nm and will always be constrained by Abbé’s limit, in this case about 200 nm. This resolution limit indicates that it is not possible to distinguish whether there is one single or more particles within each bright spot of 200 nm. Owing to the high particles brightness and their strong dilution, we assume that mainly single particles were imaged. 

### 2.2. Biostability of Lipid-Coated ZnO Nanoparticles in Physiological Media

The efficient delivery of nanoparticles to the pathological site of interest is a crucial step to achieve an effective photodynamic therapeutic effect. In the case of injection into living systems, this could be hindered by aggregation of the ZnO NPs in contact with plasma fluids: several studies have indeed shown that both circulation time in the blood stream and cellular uptake are strongly influenced by the nanoparticles’ colloidal stability in these media [20,21].

**Figure 4:**
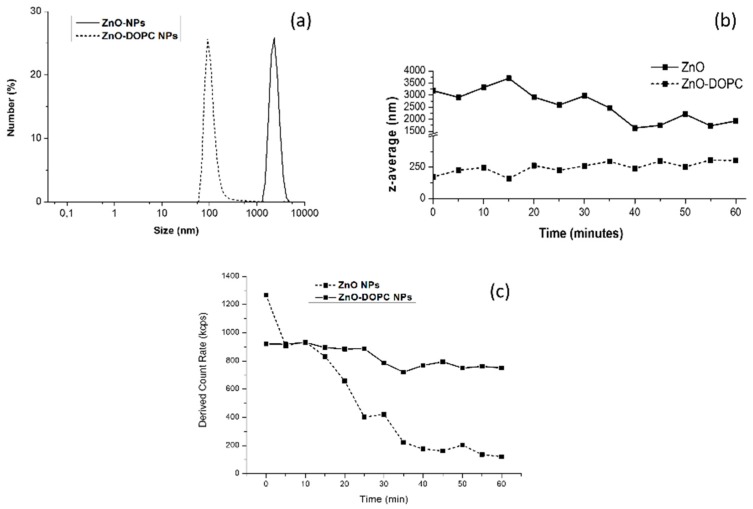
The lipid-shell increases colloidal stability of ZnO NPs in PBS: (**a**) Dynamic Light Scattering (DLS) measurements in number percent of the bare (ZnO) and lipid-coated (ZnO-DOPC) nanoparticles in Phosphate Buffered Saline (PBS); (**b**) mean hydrodynamic size (Z-average) of bare ZnO NPs (solid curve) and lipid-coated ZnO NPs (dotted curve) in PBS over time; and (**c**) derived count rate of the two samples over time.

As a first step towards the comprehension of the behavior of ZnO NPs in biological media, further Dynamic Light Scattering (DLS) experiments were performed in Phosphate Buffered Saline (PBS). Moreover, the effect of the lipid-coating on the colloidal stability of ZnO NPs has been evaluated. As shown in Figure 4a, a striking difference between the behavior of the two samples was noted when suspended in PBS. Bare ZnO NPs showed a strong aggregation behavior, forming micrometer-scale aggregates, while lipid-coated ZnO NPs did not show any aggregation, confirming the hydrodynamic size obtained in water suspension.

To further study this behavior, the mean hydrodynamic radius (z-average) of ZnO and ZnO-DOPC NPs was recorded in real-time for 1 h (Figure 4b). As soon as bare ZnO NPs were exposed to the PBS solution (time 0), the measured z-average was relatively high (3200 nm), confirming that this sample promptly formed huge aggregates. Over time, the z-average moderately decreases (down to 1890 nm), suggesting that the NPs maintain their aggregated form and partially precipitate. The marked decrease of the derived count rate over time (Figure 4c) strongly supports this hypothesis. On the contrary, lipid-coated ZnO NPs did not form micrometer-scale aggregates over time when suspended in PBS, maintaining a Z-average size between 100 and 250 nm, thus confirming their higher colloidal stability compared to the bare ZnO NPs.

The same improvement of colloidal and chemical stability of lipid-coated ZnO nanoparticles in biological media was studied in detail in a previous work [22]. As already observed for biostability assays performed in cell culture media (EMEM) and simulated human plasma (Simulated Body Fluid, SBF), this improved colloidal behavior can be attributed to the lipid shell stabilization, shielding the ZnO NPs. In particular, for PBS, the phosphate ions contained in large quantity in the buffer solution are strongly reactive towards ZnO leading to the formation of poorly soluble zinc-phosphate precipitates [23]. Thus, these data suggest that the lipid coating can limit the contact of solution’s components with the ZnO surface, preventing NPs’ aggregation.

### 2.3. Reactive Oxygen Species Generation

#### 2.3.1. ROS Generation in the Absence of External Actuation

In photodynamic therapy, it is crucial to avoid the activation of photosensitizer in the absence of light irradiation that would lead to uncontrollable and dangerous side effects. To investigate this possibility, the ROS generation by pristine ZnO nanoparticles was first studied without external actuation (ambient light) in different water and physiological media. The formation of hydroxyl and superoxide anion radicals by ZnO NPs was characterized using the Electron Paramagnetic Resonance (EPR) technique coupled with the DMPO (5,5-dimethyl-pyrroline *N*-oxide) spin trap. This compound can trap both hydroxyl and superoxide anion radicals, thus suitable for studying ROS generation by NPs. A suspension of ZnO NPs (500 μg/mL) in the tested medium was kept at 37 °C for 1 h under continuous stirring, inserted into the EPR cavity, as described in detail the Materials and Methods Section, and the EPR spectrum was recorded.

**Figure 5:**
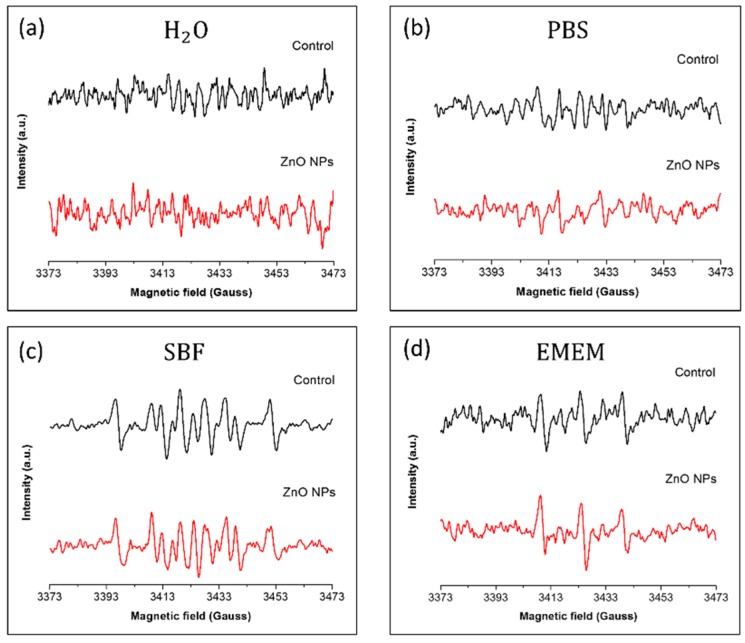
EPR spectra bare ZnO NP suspensions (500 μg/mL) in different media using DMPO (50 mM) as a spin trap: (**a**) Water; (**b**) Phosphate Buffered Saline (PBS); (**c**) Simulated Body Fluid (SBF); and (**d**) Eagle’s Minimum Essential Medium (EMEM). Black spectra: medium without ZnO NPs, Red spectra: medium with 500 μg/mL ZnO NPs.

In Figure 5, the EPR spectra corresponding to the tested suspensions in the absence (black curve) and in the presence (red curve) of pristine ZnO NPs are shown. Using water as dispersing medium, no spin adduct was detected in the obtained spectra as shown in Figure 5a. The same type of noisy signals was obtained using Phosphate Buffered Saline (PBS) as medium (Figure 5b). On the contrary, signals composed by several spin adducts were detected in Simulated Body Fluid (SBF) suspensions, as shown in Figure 5c. The presence of the ZnO nanoparticles in the medium did not modify the recorded EPR spectrum, thus indicating that the detected spin adducts were not due to ROS generation by ZnO NPs themselves, but to the reaction of the DMPO spin trap with medium components. Indeed, the high concentration of metal ions in SBF can react with DMPO forming EPR-detectable compounds [24]. Finally, when EPR spectra were recorded using the cell culture medium (EMEM), a triplet signal was detected in both control and ZnO NPs experiments. Similar to the SBF experiments, this signal is due to the reaction of medium components with the DMPO spin trap. Cell culture medium, indeed, includes several autoxidizable compounds, such as the amino-acids L-tyrosine and L-triptophan, that can oxidize the DMPO spin trap giving rise to spin adducts detectable by EPR [25].

Taken together, these measurements demonstrate that, in the absence of any external actuation such as UV irradiation, our pristine ZnO NPs cannot generate hydroxyl and superoxide anion radicals when suspended in different physiological media. This is the first step towards the synthesis of an effective photosensitizer able to work as a remotely-activated ROS generator.

#### 2.3.2. ROS Generation under UV Illumination by BARE ZnO Nanoparticles

To evaluate the capability of ROS generation by our pristine ZnO nanoparticles under UV irradiation, water suspensions of bare ZnO NPs were irradiated with UV light for 5 min and, using DMPO as a spin trap, the EPR spectrum was recorded as described in the Materials and Methods Section.

**Figure 6:**
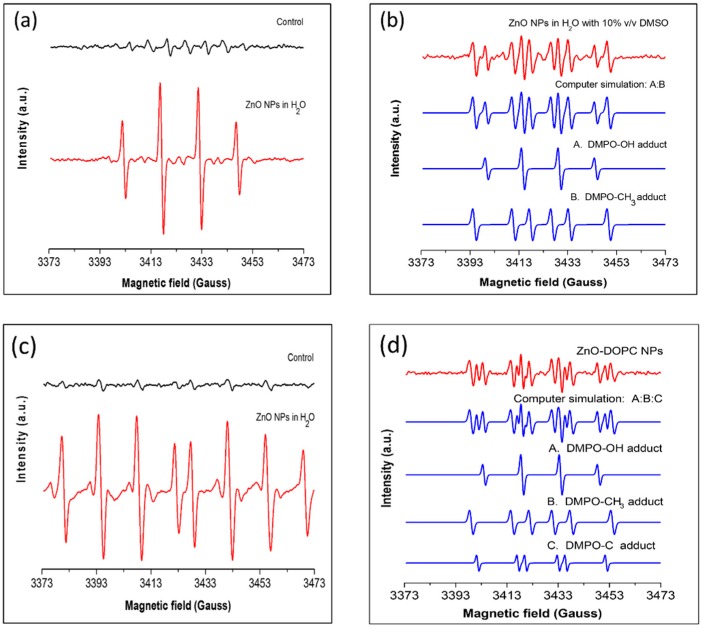
ROS formation in aqueous suspensions of bare and lipid-coated ZnO nanoparticles (500 μg/mL) irradiated with UV light. (**a**) EPR spectrum obtained after the irradiation with UV light of an aqueous solution with (red) and without (black) ZnO NPs using DMPO as spin trap. The characteristic four peaks of the DMPO-OH adduct are detected. (**b**) Effect of the addition of 10% *v*/*v* Dimethyl Sulfoxide (DMSO) to the solution. New peaks corresponding to the DMPO-CH_3_ spin adduct are detected (red curve), as confirmed by the computer simulation of the experimental spectrum (blue curves). (**c**) Verification of the absence of superoxide anion radical generation using DEPMPO as spin trap. Only the characteristic peaks of DEPMPO-OH spin adduct are detected with (red) and without (black) ZnO NPs in suspension. (**d**) Effect of surface functionalization on ROS generation by ZnO NPs under UV illumination detected using DMPO as spin trap. Using lipid-shell functionalized ZnO NPs, a complex EPR spectrum is obtained (red curve). Computer simulation reveals that, together with the detection of the DMPO-OH and DMPO-CH_3_ spin adducts, a short-chain carbon-centered radical is detected (blue curves).

As shown in Figure 6a, a characteristic DMPO-OH spin adduct giving rise to four resolved peaks was obtained when ZnO NPs were present in the water solution. The control experiment, performed in the absence of particles, gave rise to far smaller DMPO-OH signals due to water photolysis. These signals suggest that our pristine ZnO nanoparticles greatly enhanced the generation of hydroxyl radicals under UV illumination in water.

Since DMPO can also trap superoxide anion radicals to produce the spin adduct DMPO-OOH, which is not stable and readily decomposes to the DMPO-OH adduct [26,27], O_2_· generation could not be excluded. To determine whether the spectrum presented in Figure 6a was due to the generation of superoxide and/or hydroxyl radical, DMSO was added to the ZnO water suspension to scavenge the photo-generated hydroxyl radical. The resulting EPR spectrum is shown in Figure 6b. The addition of DMSO significantly reduced the DMPO-OH quartet signal, thus suggesting that OH· radicals are actually generated by ZnO nanoparticles. Computer simulations were performed to identify the new peaks present in the detected signal (blue curves). The simulated spectrum matched well with the experimental one: together with the DMPO-OH adduct, the DMPO-CH_3_ adduct was detected. This is formed by the reaction of the photo-generated OH radical with the DMSO molecule [28], thus further confirming the hydroxyl radical generation.

**Figure 7:**
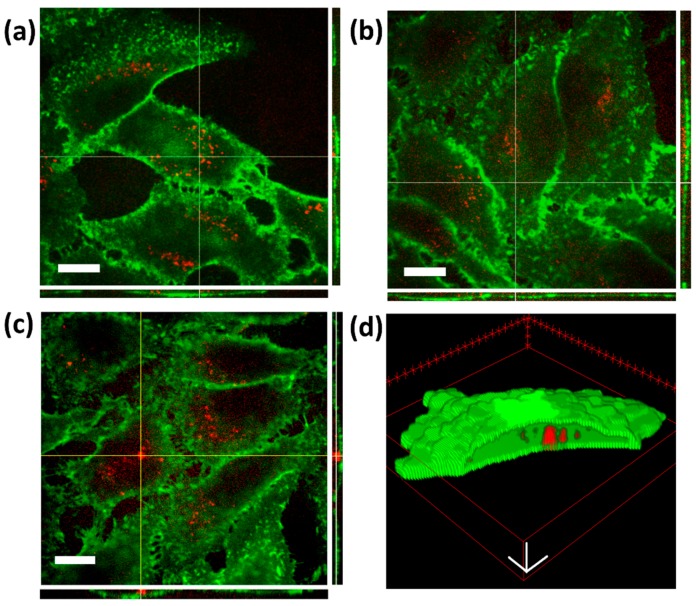
Lipid-shell functionalized ZnO NPs are successfully internalized by HeLa cells after 24 h incubation. (**a**–**c**) Representative fluorescent image of HeLa cells (membranes in green) incubated for 24 h with fluorescent ZnO-DOPC NPs (in red). Rectangles on the right side and bottom of each image show orthogonal views of the z-stack (along the yellow lines) of images proving that the particles are inside the cells. Scale bar: 10 µM. (**d**) 3D representation of ZnO-DOPC nanoparticles taken up into the HeLa cell. ZnO-DOPC NPs (in red) are clearly visible inside the cellular membrane (in green). Z-stacks images were processed with Particle_in_Cell-3D Macro [30] using Fiji software. Scale bar: 5 µM.

To verify the absence of the superoxide anion (O_2_), the dedicated spin trap DEPMPO was used. This can trap hydroxyl and superoxide anion radicals forming radical-specific stable spin adducts, thus enabling the direct detection of the superoxide anion [29]. In Figure 7, the EPR spectra obtained after UV illumination of water suspensions are shown. In the presence of ZnO NPs in solution (red curve), eight resolved peaks were detected, corresponding to the characteristic peaks of the DEPMPO-OH spin adduct. These were also detected in the control experiment, but with much lower intensities, and are thus attributed to water photolysis. The absence of the DEPMPO-O_2_·spin adduct strongly suggests that superoxide anion radicals are not photo-generated by ZnO NPs.

#### 2.3.3. Effect of Surface Functionalization on ROS Generation

Since the photo-generation of an electron–hole pair and the subsequent formation of hydroxyl radicals happens at the surface of the ZnO nanoparticle, it can be expected that modifying the chemistry of the surface, i.e., by phospholipid bilayer coating, would influence its ROS generation ability. To investigate this hypothesis, EPR spin-trapping experiments were performed using lipid-coated ZnO nanoparticles and DMPO as spin trap. Water suspension of ZnO-DOPC NPs were irradiated with UV for 5 min and the EPR spectrum recorded.

As shown in Figure 6d, a far more complex EPR spectrum was recorded compared to the spectrum obtained for bare ZnO NPs (Figure 6a, red curve). Indeed, the new spectrum is a composition of several DMPO spin adducts. To distinguish the different spin adducts, computer simulation was performed to fit the experimental spectrum with characteristic DMPO adducts spectra. The simulated spectra (blue curves) matched well with the experimental one (red curve), and the single contributions could be distinguished. Indeed, together with the DMPO-OH spin adduct, DMPO-CH_3_ and a short-chain carbon-centered radical adduct were detected. Importantly, the presence of the DMPO-OH spin adduct in the experimental spectrum suggests that phospholipid bilayer coating did not prevent the hydroxyl radical photo-generation. The other two spin adducts derive from the reaction of DMPO with short-chain carbon-centered radicals probably formed by fragmentation of the phospholipid chain undergoing lipid peroxidation. Indeed, this could be initiated by the oxidative attack of the photo-generated hydroxyl radicals that, being highly reactive species, can oxidize unsaturated fatty acids in lipid membranes starting the lipid peroxidation process [31].

Together, these results confirm the possibility to use the lipid-coated ZnO NPs as nano ROS-generator under UV irradiation and reveal that the surface functionalization can clearly influence the type of photo-generated ROS.

### 2.4. Cellular Uptake and Intracellular ROS Generation 

#### 2.4.1. Uptake and Internalization Pathway in Cancer Cells

In photodynamic therapy, the internalization of the photosensitizer by the target malignant cells is a crucial step toward an effective therapeutic effect. ROS have short lifetimes and diffuse poorly in biological environment: therefore, they can be effective in exerting a cytotoxic effect only if photo-generated intracellularly [32].

To determine if cancer cells could effectively internalize lipid-coated ZnO NPs, human epithelial carcinoma cells (HeLa) were used as target cells. Cancer cells were treated with 18 μg/mL of ZnO-DOPC NPs and after 24 h of incubation the cellular uptake was qualitatively assessed by fluorescence images using Spinning Disk Microscopy, as shown in Figure 7. Z-stacks of live-cell images allowed the direct visualization of the lipid-coated nanoparticles localized inside the cellular membrane, thus confirming the successful uptake.

**Figure 8:**
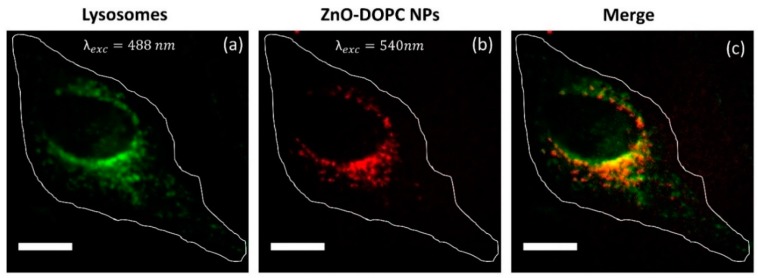
Cancer cells internalized lipid-coated ZnO NPs through an endosomal-lysosomal pathway. Wide-Field Fluorescence images of: (**a**) Lysosomes marked with CellLight Lysosomes-GFP; (**b**) Lipid-coated ZnO NPs marked with Atto550-NHS ester; and (**c**) The merged images showing the co-localization of the ZnO-DOPC NPs with the intracellular lysosomes. Scale bar: 5 µM.

Several studies demonstrated that, in addition to an efficient cellular uptake, the intracellular localization of the photosensitizer can greatly affect the therapeutic outcome of photodynamic therapy. ROS have a short life-time and react close to their site of generation, therefore the photodamage can depend on the precise subcellular localization of the NPs [32]. Thus, a careful investigation of the intracellular localization of our lipid-coated ZnO NPs was performed by fluorescent microscopy co-localization experiments using a fluorescent lysosomal marker, as described in detail in the Materials and Methods Section and shown in Figure 8. Co-localization of the lysosomal fluorescent spots (red) with the ZnO-DOPC NPs ones (green) suggests that the cancer cells internalized the lipid-coated ZnO NPs through an endosomal-lysosomal pathway, which eventually led to the internalization of the nanoparticles inside the cancer cell lysosomes (Figure 8c).

#### 2.4.2. Intracellular ROS Generation

**Figure 9:**
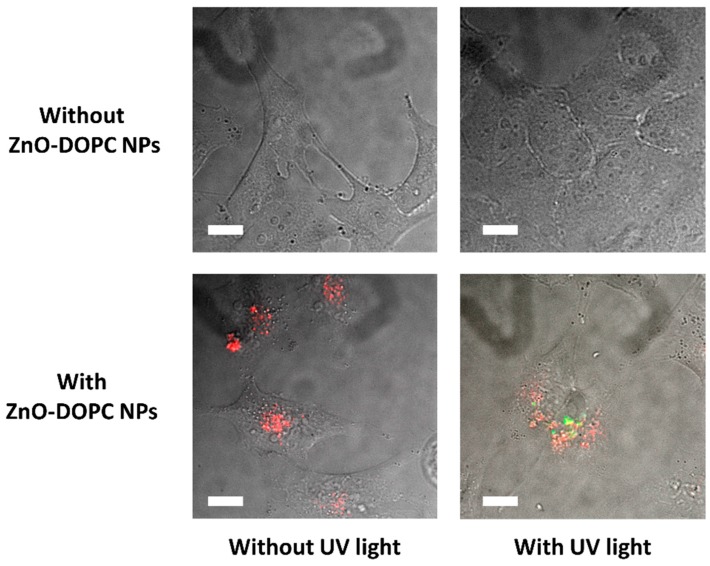
Intracellular ROS generation by lipid-coated ZnO nanoparticles. Scale bar: 5 µM.

Finally, to evaluate if the lipid-coated ZnO NPs preserved their ability to photo-generate ROS after their internalization inside cancer cells, fluorescence microscopy experiments based on the oxidation of 2′-7′-dichlorofluorescein (DCF) were performed. HeLa cells were incubated with DCF and lipid-coated ZnO NPs, and after 24 h irradiated with UV light. As shown in Figure 9, the control experiments did not show any green fluorescence due to DCF oxidation, suggesting that the exposure of cancer cells to the UV irradiation did not generate any ROS in the absence of ZnO-DOPC NPs. On the contrary, when HeLa cells were incubated with ZnO-DOPC NPs (red spots) and exposed to UV light for 30 s, green fluorescence due to the oxidation of DCF appeared close to the internalized NPs. This indicates that lipid-coated ZnO nanoparticles were able to generate ROS inside cancer cells when irradiated with UV light, thus suggesting ZnO-DOPC NP as innovative ROS-generator agent for photodynamic therapy.

### 2.5. Preliminary Nanoparticles Cytotoxicity and Photodynamic Effect Study

Cytotoxicity and photodynamic effect of both bare ZnO and lipid-coated ZnO nanoparticles were investigated on HeLa cells. It is worth mentioning that, in coherence with both the internalization and the intra-cellular ROS generation ability experiments, amine-functionalized ZnO nanoparticles were used in the experiments (EPR and Z-potential measurements of amine-functionalized ZnO NPs are shown in the Supplementary Materials).

**Figure 10:**
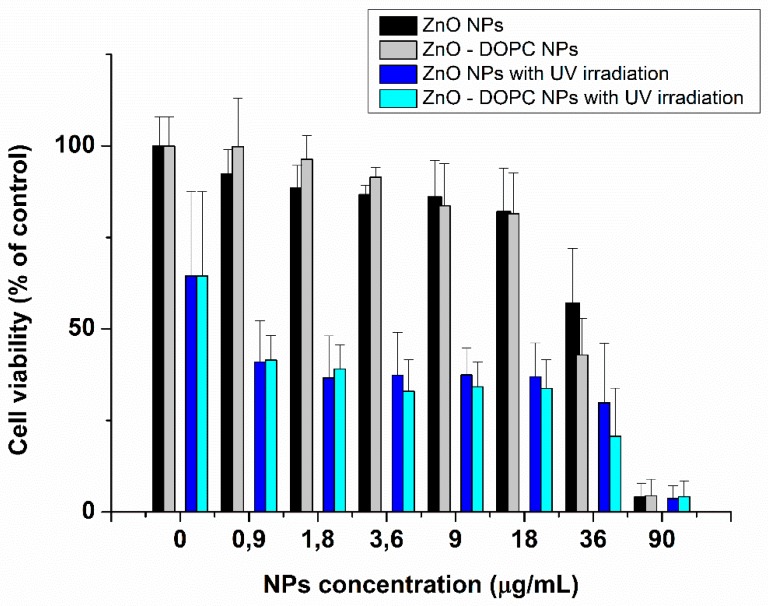
Cytotoxicity and photodynamic effect of different concentrations of ZnO nanoparticles and lipid-coated ZnO nanoparticles with and without UV irradiation (30 s).

The effects of different concentrations of ZnO and ZnO–DOPC nanoparticles on HeLa cell culture for 24 h are shown in Figure 10. From a quantitative point of view, these data confirm the cytotoxic behavior (in absence of UV light activation) of both ZnO and ZnO-DOPC NPs only at high NPs concentration. The two kinds of ZnO NPs had no visible cytotoxic effect up to a concentration of about 18 μg/mL, while both showed a significant cytotoxic effect for higher concentrations. 

Moreover, HeLa cell cultures were irradiated with UV light (at a wavelength of 255 nm) for 30 s to preliminary evaluate the ability of NPs-induced photo-generated ROS to induce cytotoxic effects in vitro. Cancer cells treated with both kind of ZnO NPs showed a marked decrease of cell viability after 24 h from the UV-exposure, compared to the control without NPs-treatment (first columns of the graph in Figure 10 reporting the concentration of 0 µg/mL of nanoparticles). These preliminary data suggest that ZnO, used at non-toxic concentrations, can induce cytotoxic effects when irradiated with UV light. Moreover, the lipid-coating, while increasing NPs stability in biological media, does not decrease its photodynamic therapeutic effect. Future studies will focus on the optimization of the light exposure parameters to enhance the cancer cell killing photodynamic efficacy of ZnO-DOPC NPs.

## 3. Materials and Methods

### 3.1. Synthesis and Functionalization of ZnO Nanoparticles

Zinc oxide nanoparticles were prepared by applying a similar wet chemical method as that described in the previous report [33]. Zinc acetate dihydrate (Zn(CH_3_COO)_2_·H_2_O (3.73 mmol) was dissolved in methanol (42 mL) and heated under continuous stirring. As the temperature reached 60 °C, 318 μL of bi-distilled water and a solution containing 7.22 mmol of NaOH in 23 mL of methanol were added drop by drop to the zinc acetate solution. This was maintained at 60 °C for 2.15 h and then washed two times with fresh ethanol centrifuging at 3046 rcf for 5 min.

ZnO NPs were functionalized according to previously reported method [34]. In brief, the amino-propyl functionalized zinc oxide nanoparticles (ZnO-NH_2_ NPs) used for fluorescence microscopy experiments were obtained exploiting the reaction between bare ZnO nanoparticles and 3-aminopropyltrimethoxysilane (APTMS), while the lipid-coated zinc oxide nanoparticles (ZnO-DOPC NPs) were prepared by a solvent exchange method using the commercial phospholipid DOPC (1,2-dioleoyl-sn-glycero-3-phosphocholine), as described in previous reports [35].

### 3.2. Characterization of Zinc Oxide Nanoconstructs

The morphology of ZnO and ZnO-DOPC nanoparticles was studied by Field Emission Scanning Electron Microscopy (FESEM, Auriga and Merlin, Karl Zeiss, Oberkochen, Germany). The diluted samples were spotted on a silica wafer and coated by a thin layer of Pt for further imaging. The particle size and Zeta potential of the three samples were determined using the Dynamic Light Scattering (DLS) technique (Zetasizer Nano ZS90, Malvern, Worcestershire, UK), while the crystalline structure was analyzed by X-ray diffraction with a X’Pert diffractometer in configuration θ–2θ Bragg-Brentano using a Cu-Kα radiation (λ = 1.54 Å, 40 kV and 30 mA).

To confirm the formation of the supported lipid bilayer on the surface of ZnO-DOPC nanoparticles, fluorescence co-localization experiments were performed. The DOPC shell was labeled with 1% Bodipy-DHPE lipid by incubating this dye (0.2 µg per mg of lipids) with the dispersed DOPC lipids prior to assembly. ZnO nanoparticles, after amine functionalization using APTMS, were labeled with Atto550-NHS ester dye (2 µg per mg of NPs) overnight under stirring at RT and then washed twice with fresh ethanol. A fully-motorized wide-field inverted microscope Nikon Eclipse TiE (Nikon, Tokyo, Japan), in combination with a high resolution sCMOS camera (Zyla 4.2 Plus from Andor) and an immersion 60× oil objective was used.

### 3.3. Bio-Stability Assay

For the bio-stability assay, bare and lipid-coated ZnO nanoparticles were tested in Phosphate Buffered Saline (PBS, Sigma Aldrich, St. Louis, MO, USA). Dynamic Light Scattering (DLS) measurements were performed suspending 500 μg of nanoparticles in 1 mL of Phosphate Buffered Saline (PBS).

### 3.4. Spin Trapping Measurements Coupled with EPR Spectroscopy

The EPR-spin trapping technique coupled with the spin traps 5,5-dimethyl-l-pyrroline-*N*-oxide (DMPO, 50 mM) and 5-(diethoxyphosphoryl)-5-methyl-l-pyrroline-*N*-oxide (DEPMPO, 40 mM) (Sigma, St. Louis, MO, USA) was used to detect hydroxyl radicals and superoxide radicals.

To verify the ROS generation by bare ZnO NPs (500 μg/mL) in different biological media, 10 μL of 1 M DMPO were mixed with 190 μL of the various tested media (water, PBS, Simulated Body Fluid (SBF) or Cell Culture Media (EMEM)) containing ZnO NPs. The resulting solution was maintained under continuous stirring (150 rpm) at constant temperature of 37 °C for 1 h and transferred into a quartz microcapillary tube for the EPR measurement. For the detection of UV-induced ROS generation, 100 μL of the selected spin trap were mixed with 100 μL of the appropriate ZnO NPs suspension. The solution was mixed with a vortex, irradiated with UV light (wavelength 350–450 nm, Intensity: 150 mW/cm^2^) for 5 min and then promptly transferred into a quartz microcapillary tube. In all EPR measurements, 25 μM of Diethylenetriaminepentaacetic acid (DTPA) was added to the solution as metal ion chelator. For experiments labeled as “Absence of external actuation”, there was an ambient light illumination.

The microcapillary tubes were then inserted in the EPR cavity and the spectra were recorded on a Bruker EMXnano X-Band spectrometer (Bruker, Billerica, MA, USA). The EPR measurement conditions were as follows: Frequency, 9.74 GHz; scan width, 100 G; receiver gain, 60 dB; time constant, 1.28 ms; sweep time, 160 s; scans, 10.

After acquisition, the spectrum was processed using the Bruker Xenon software (Bruker, Billerica, MA, USA) for baseline correction. Simulation of the recorded spectra was performed using the Bruker SpinFit software.

### 3.5. Internalization Experiments in HeLa Cells

HeLa cells were cultured in Dulbecco’s Modified Eagle Medium (DMEM) with 10% FBS and 1% PenStrep. They were seeded into ibiTreat µ-slides (ibidi) at a concentration of 5000 cells per well in 300 µL of DMEM. The day after seeding, cells were incubated with particles for 24 h and for the lysosome experiments also with 8 µL of CellLight Lysosomes-GFP (Thermo fisher scientific, Waltham, MA, USA). The particles were labeled with Atto633-NHS overnight and then prepared as for the characterization described above. For internalization experiments cells were stained with WGA488 (Thermo fisher scientific, Waltham, MA, USA), and washed with DMEM prior to imaging. For imaging we used a spinning disk microscope (Zeiss Cell Observer SD with a Yokogawa spinning disk unit CSU-X1). Lysosome-GFP and WGA were excited with a 488 nm laser and the particles with a 639 nm laser. Band-pass filters 525/50 and 690/60 (both Semrock) were used in the detection path for Lysosome-GFP/WGA and the particles, respectively.

### 3.6. Detection of Intracellular ROS Generation

HeLa cells were cultured, seeded and incubated with particles as described above for the internalization experiments. 2′,7′-dichlorofluorescein diacetate (DCFDA) was dissolved in DMSO shortly before use and added to the cells yielding a final concentration of 0.13 µM. After an incubation time of 30 min cells were imaged to obtain the control before UV illumination. After 30 s of UV illumination and another 30 min incubation, cells were imaged for the UV-illumination. Microscopy was performed as for the internalization experiments and a 488 nm laser and a BP 525/50 filter were used to image the DCFDA. 

### 3.7. Cytotoxicity and Photodynamic Experiments

HeLa cells were seeded at a concentration of 5000 cells per well into 96 well plates (Corning) containing a final volume of 100 mL of medium. One day after seeding they were incubated with NPs at the desired concentration. Twenty-four hours after incubation with NPs, MTT assay were performed according to the standard protocol.

For photodynamic experiments, after 5 h of incubation with the desired NP concentrations, cells were exposed to 30 s of UV illumination and MTT were carried out after 24 h to UV exposure.

## 4. Conclusions

In the present study, we propose the synthesis and characterization of lipid-coated ZnO nanoparticles as new photosensitizer for PDT against cancer. First, we show that the lipid-coating increases the colloidal stability of the ZnO NPs in Phosphate Buffered Saline (PBS). Then, we demonstrate that bare and lipid-coated ZnO nanoparticles generate hydroxyl radicals only when irradiated with UV light. Moreover, the phospholipid bilayer coating induces the photo-generation of short-chain carbon centered free radicals, thus suggesting that the nanoparticle surface chemistry plays a crucial role in determining the type of photo-generated free radicals. Finally, we show that lipid-coated NPs are effectively internalized by HeLa cells through an endosomal-lysosomal pathway and that they can generate ROS even once internalized and kill cancer cell at non-toxic concentration thanks to the UV-stimuli activation.

The herein reported results pave the way for a more conscious design of nanoparticles for PDT treatment, with the surface chemistry being an important factor to be considered for an efficient ROS production, and imply the potential of lipid-coated NPs as innovative ROS-generators for therapeutic activity against cancer.

## Supplementary Materials

The following are available online at http://www.mdpi.com/2079-4991/8/3/143/s1, Figure S1: ROS formation in aqueous suspensions of amine-functionalized ZnO nanoparticles (500 μg/mL) irradiated with UV light). Computer simulation reveals that DMPO-OH and DMPO-CH_3_ spin adducts are detected (blue curves). Figure S2: Z-potential measurement of pristine ZnO NPs and amine-functionalized NPs.
