# Supplementary material for: Lipid-Coated Zinc Oxide Nanoparticles as Innovative ROS-Generators for Photodynamic Therapy in Cancer Cells
Source: Nanomaterials (Basel). 2018 Mar 2;8(3):143. doi: 10.3390/nano8030143 (PMC5869634; doi:10.3390/nano8030143)
Supplement: Supplementary file 1 [file nanomaterials-08-00143-s001.pdf]

Supplementary Information

Lipid-coated zinc oxide nanoparticles as innovative ROS-generators for photodynamic therapy in cancer cells

Andrea Ancona [1], Nadia Garino [1,3], Bianca Dumontel[1] Benjamin Demarco[2], Dimitra Chatzitheodoridou[2], Walter Fazzini[1], Hanna Engelke[2] and Valentina Cauda[1, 3, *]

[1] Department of Applied Science and Technology, Politecnico di Torino, Corso Duca degli Abruzzi 24, 10129 Turin, Italy;
[2] Department of Chemistry, Ludwig-Maximilians-University of Munich, Butenandtstrasse 11E, 81377 Munich, Germany;
[3] Center for Sustainable Future Technologies - CSFT@POLITO, Istituto Italiano di Tecnologia, Corso Trento 21, 10129 Turin, Italy;
* Correspondence: valentina.cauda@polito.it; Tel.: +39-011-090-7389

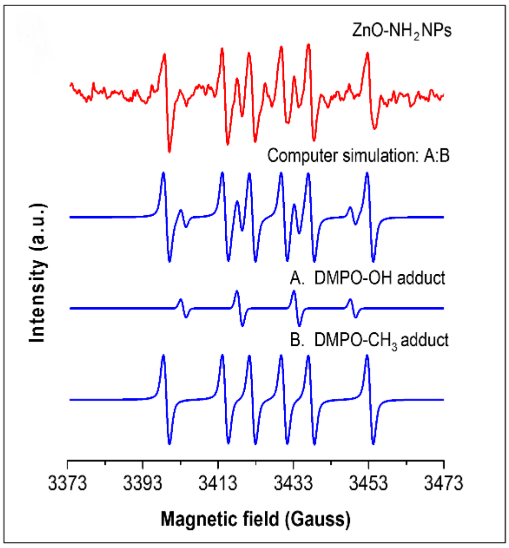

Figure S1. ROS formation in aqueous suspensions of amine-functionalized ZnO nanoparticles (500 µg/ml) irradiated with UV light). Computer simulation reveals that DMPO-OH and DMPO-CH_3 spin adducts are detected (blue curves).

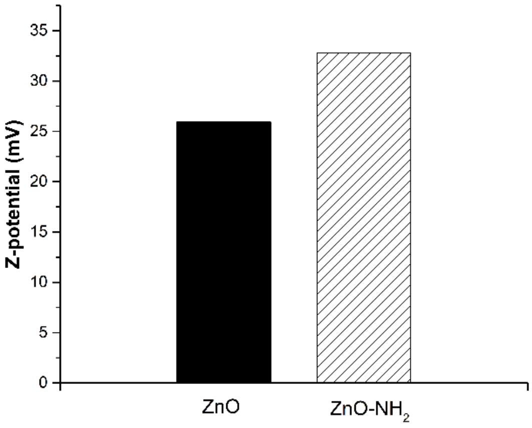

Figure S2. Z-potential measurement of pristine ZnO NPs and amine-functionalized NPs.
